# The Impact of Preoperative Invasive Nodal Staging on Unexpected Mediastinal Upstaging in Early-Stage Non-small Cell Lung Cancer

**DOI:** 10.1245/s10434-025-17034-0

**Published:** 2025-02-20

**Authors:** Kian C. Banks, Varada Sarovar, Angela Sun, Rachel K. Wile, Katherine E. Barnes, Jeffrey B. Velotta

**Affiliations:** 1 Division of General Surgery, Department of Surgery, University of California San Francisco East Bay, Oakland, CA USA; 2 Division of Research, Kaiser Permanente Northern California, Pleasanton, CA USA; 3 University of California, Berkeley, CA USA; 4 University of California San Francisco School of Medicine, San Francisco, CA USA; 5 Division of Thoracic Surgery, Department of Surgery, Kaiser Permanente Oakland, Oakland, CA USA; 6 Department of Clinical Science, Kaiser Permanente Bernard J. Tyson School of Medicine, Pasadena, CA USA

**Keywords:** Early-stage, Non-small cell lung cancer (NSCLC), Endobronchial ultrasound (EBUS), Practice guidelines, Invasive nodal staging, Mediastinoscopy, Mediastinal nodal disease, Stage change, Unexpected N2

## Abstract

**Introduction:**

Preoperative invasive nodal staging is standard of care for early-stage non-small cell lung cancer (NSCLC). Complications and delays in care are not negligible and diagnostic accuracy varies. In our system, invasive nodal staging is performed for clear radiographic indications (node > 1.0 cm short axis or standardized uptake value > 3.0, tumor > 4.0 cm). This study assessed whether unexpected mediastinal upstaging was less common in patients receiving preoperative invasive nodal staging.

**Methods:**

This retrospective study evaluated nodal upstaging, defined as pathological N2 or IIIA+ disease, based on receipt or non-receipt of invasive nodal staging. Clinical stage I–II NSCLC patients who underwent resection (2009–2019) were identified from our cancer registry. Stage and preoperative nodal staging information were confirmed through chart review. Associations between patient characteristics, invasive nodal staging receipt, and clinical to pathological stage changes were analyzed.

**Results:**

Among 2576 patients, 18.7% (*n* = 481) underwent invasive nodal staging. After resection, 6.2% of all patients had nodal upstaging and 24.9% had TNM upstaging. Only 0.3% (*n* = 9) were upstaged to N2 and 0.5% (*n* = 13) were upstaged to IIIA+. Lack of preoperative nodal sampling was not associated with N2 or IIIA+ upstaging. Findings were consistent in subanalyses of patients with surgical specimens meeting Commission on Cancer nodal sampling criteria and with clinical IB+ disease.

**Conclusions:**

Although most patients did not undergo invasive nodal staging, <1% had unexpected N2 on surgical pathology. There was no association between lack of preoperative invasive nodal sampling and N2 nodal upstaging. Preoperative invasive nodal staging did not increase pathologic N2 nodal upstaging in early-stage NSCLC patients in our integrated health system.

**Supplementary Information:**

The online version contains supplementary material available at 10.1245/s10434-025-17034-0.

Accurate risk stratification improves non-small cell lung cancer (NSCLC) survival.^1^ Nodal status, which is most reliably defined by surgical pathology, is an important prognosticator for NSCLC; however, the role of preoperative nodal status in treatment algorithms for surgically fit patients is still evolving. According to National Comprehensive Cancer Network (NCCN) and American College of Chest Physician (ACCP) guidelines, the majority of surgical candidates have an indication for invasive nodal staging,^2^ but fewer than half of patients in the US actually undergo the recommended invasive staging.^3^

The optimal staging work-up for early-stage NSCLC remains unclear. For peripheral cancers < 3 cm that appear node-negative on computed tomography (CT) and positron emission tomography (PET)/CT, there is consensus that invasive nodal staging is optional due to the low likelihood of positive mediastinal lymph nodes.^4,5^ For node-positive disease on imaging (CT or PET/CT), there is also international consensus that thorough invasive nodal staging is necessary, either with endobronchial ultrasound (EBUS) or mediastinoscopy. However, in clinical practice, there is dissent over invasive nodal staging for imaging-based node-negative tumors that are > 3 cm or central, illustrated by the fact that providers do not follow invasive nodal staging guidelines the majority of the time.^3^ The additional step may cause treatment delays and appropriate risk stratification may be achieved in a more streamlined manner through high-quality systematic nodal sampling during surgery in appropriately selected patients.

Studies on the diagnostic accuracy of PET/CT, EBUS, and mediastinoscopy are remarkably heterogeneous, and the expansion of endoscopic techniques has led to further variation in results and recommendations.^6,7^ Invasive nodal staging procedures are highly operator-dependent and can only access a specific set of nodes.^8^ Historically, preoperative lymph node staging predominantly served to rule out mediastinal lymphatic involvement because N2 disease changed management, particularly resectability. However, evolving definitions of N2 resectability, neoadjuvant therapy considerations, and the ability of EBUS to obtain a tissue diagnosis and stage nodes simultaneously have made invasive nodal staging conversations far more complex. Specifically, a growing body of evidence demonstrates limited N2 (IIIA) disease may benefit from resection with systemic therapy.^9^ In the immunotherapy era, optimal treatment sequencing (neoadjuvant or adjuvant) is unclear, particularly for early-stage disease.^10^ EBUS and mediastinoscopy are treated as interchangeable modalities in NCCN guidelines, yet they can access different nodal stations with different stage-dependent false-negative rates and unique risk profiles, and well-trained proceduralist availability varies widely.^8,11–15^ EBUS can be combined with diagnostic procedures, while mediastinoscopy is commonly performed under the same anesthetic procedure as therapeutic resection and results are subsumed within pathologic staging.

Complex considerations notwithstanding, the proportion of imaging-based early-stage patients who undergo invasive procedures with negative results, either as true or false negatives, has received inadequate attention. There may be a role for more tailored recommendations for routine preoperative invasive nodal staging for early-stage NSCLC. Post-procedural complications, increased anesthesia time, and delays in care associated with coordinating additional procedures or managing complications are not negligible.^12,16^ Invasive nodal staging has been bluntly applied to imaging-based early-stage NSCLC and may demand greater precision to increase the pretest probability of positive nodes with invasive staging.

In our large, integrated health system, we apply somewhat narrower invasive nodal staging criteria and significant clinical gestalt is allowed. We sought to assess whether patients who received preoperative invasive nodal staging had less clinical to pathologic stage change compared with those who did not. The primary objective was to determine whether receipt or non-receipt of invasive nodal staging was associated with increased unexpected N2 or IIIA disease on surgical pathology, particularly with regard to upfront resection.

## Methods

This data-only, multicenter, retrospective study was approved by the Kaiser Permanente Northern California (KPNC) Institutional Review Board (IRB) for the Protection of Human Subjects with a waiver of individual informed consent (IRB# 1610759). This study was funded by a Permanente Medical Group Delivery Science grant. The funding source had no role in the study design; data collection, analysis or interpretation; manuscript authorship; or publication decisions.

### Study Population

Adult patients with an incident diagnosis of clinical stage I–II NSCLC who underwent elective surgical resection with curative intent at KPNC between 2009 and 2019 were identified using institutional cancer registry and electronic health record (EHR) databases. Patients who received neoadjuvant chemotherapy, lacked appropriate preoperative imaging (defined as a chest CT and/or PET/CT within 6 months of surgery), were over 85 years of age, or had less than 1 year of continuous KPNC health plan membership before surgery were excluded.

### Setting

KPNC is an integrated healthcare delivery system with 21 hospitals that provides healthcare to 4.4 million members, nearly 30% of Northern California’s population.^17^ KPNC providers generally follow NCCN guideline recommendations for NSCLC management. However, invasive nodal staging is not routinely performed unless there is a clear radiographic indication (node >1.0 cm short axis, standardized uptake value [SUV] > 3.0, tumor > 4.0 cm [irrespective of location]) and staging is often informed by significant clinical gestalt. EBUS is limited to interventional pulmonologists at select hospitals, while mediastinoscopy is performed by general thoracic surgeons.

### Data Sources and Variables

Individual-level data on the following sociodemographic and clinical history variables were extracted, as of surgery date, from KPNC administrative and clinical databases: age, sex, race and ethnicity, neighborhood deprivation index (NDI), need for interpreter, body mass index (BMI), Charlson comorbidity index (CCI) score, smoking history, date and type of surgery, and receipt, modality, and pathology text results of preoperative invasive nodal staging. NDI was divided into ordinal quartiles, where the lowest is least deprived. BMI was categorized into < 18.5 (underweight), 18.5 to < 25 (healthy weight), 25 to < 30 (overweight) and > 30 (obese) kg/m^2^, and CCI was categorized as 0 (none), 1–2 (mild), 3–4 (moderate), and 5+ (severe). Data on tumor characteristics, including histology and American Joint Committee on Cancer (AJCC) TNM clinical and pathological stage, were extracted from the KPNC Cancer Registry, which adheres to the US Surveillance, Epidemiology, and End Results (SEER) program data standards. The corresponding AJCC staging system (version 6, 7, or 8) for the year of NSCLC diagnosis was used; consistent systems were used for both clinical and pathologic staging for a given patient.

The accuracy of extracted data on receipt of surgical resection for curative intent, clinical and pathological stage, and nodal sampling and staging procedures was verified through structured chart review. Staging discrepancies were reviewed and adjudicated by a second reviewer. For clinical TNM stage, reviewers first compared the clinician-reported clinical stage with other data in the chart, specifically the preoperative chest CT for T stage and the CT and PET/CT for N and M stages. If the patient underwent invasive nodal staging in advance of surgery, this information also informed the clinical N stage. Consistent with current reporting conventions, if preoperative nodal staging was performed on the same-day as the tumor resection, this additional histologic information only informed the pathologic stage. Pathologic TNM stage was defined as documented by the pathologist in their pathology report. Surgical procedures included wedge resection, segmentectomy, lobectomy, bilobectomy, and pneumonectomy.

Receipt of invasive nodal staging was defined by either EBUS with attempted lymph node biopsy or by mediastinoscopy after NSCLC diagnosis. If lymph node biopsy was not attempted during the EBUS, these patients were not considered to have undergone invasive nodal staging. If a patient underwent both EBUS and mediastinoscopy on the same day, the more invasive modality was reported (mediastinoscopy > EBUS). If the patient underwent invasive nodal staging on two separate occasions (e.g., EBUS followed by mediastinoscopy), only the first modality was reported. These same rules informed the timing of invasive nodal staging in relation to resection (in advance or same day).

The primary outcome of interest was ‘substantial [clinical to pathologic] upstaging’, defined as any pathologic TNM stage that would have impacted tumor resectability or treatment sequencing if known preoperatively. Cancers that were upstaged to N2 or IIIA+ after resection were considered to be ‘significantly treatment-altering’. Secondary outcomes were overall clinical to pathologic stage change (upstaged, downstaged, no change, undecided) for both nodal (N) status and TNM status. ‘Undecided’ was assigned when either the clinical or pathologic nodal status was Nx.

### Statistical Analysis

Analyses were performed using STATA v16.1 (StataCorp LLC, College Station, TX, USA). We performed analyses to evaluate the association of preoperative invasive nodal staging receipt with patient and tumor characteristics and outcomes, using the Kruskal–Wallis (continuous), Chi-square (categorical, cell *n *> 5) or Fisher’s exact (categorical, cell *n* < 5) tests as appropriate.

Given accurate pathologic staging and identification of unexpected N2 depends on adequate surgical lymph node sampling, subgroup analyses were limited to patients whose surgical specimens met the American College of Surgeons’ Commission on Cancer (CoC) nodal sampling requirements (3N2 1N1).^18^ Subgroup analyses were also limited to stage-defined patients with clinical stage IB or higher (i.e., removal of clinical IA and undecided) tumors, because invasive nodal staging is already optional for peripheral IA tumors according to NCCN and ACCP guidelines.

## Results

A total of 2586 patients with early-stage NSCLC who underwent primary resection were initially identified from our institutional cancer registry. After structured chart review, 10 patients were excluded: 7 did not undergo surgery for NSCLC with curative intent and 3 were clinical stage III or IV. Ultimately, 2576 patients were included in our analyses. The median age was 70.2 years (interquartile range [IQR] 64.1–75.8), and 59.9% were female (Table 1). The majority had at least one important comorbidity (CCI 1+: 84.1%) and a history of smoking tobacco (77.5%). Most patients had adenocarcinoma (72.7%) and clinical stage I cancer (87.7%) without known or suspected nodal involvement (cN0: 95.1%).

**Table 1:** Patient covariates by receipt of preoperative invasive nodal staging, all patients

	All [*N* = 2576]	No preoperative nodal staging [*n* = 2095]	Preoperative nodal staging [*n* = 481]	*p*-value
Sociodemographic variables				
Age	70.2 [64.1–75.8]	70.0 [64.0–75.7]	71.1 [64.3–76.5]	0.06
Female	1544 (59.9)	1294 (61.7)	250 (52.0)	< 0.001
Race/ethnicity				0.37
Asian/Pacific Islander	395 (15.3)	320 (15.3)	75 (15.6)	
Black	186 (7.2)	141 (6.7)	45 (9.4)	
Hispanic/Latinx	162 (6.3)	133 (6.4)	29 (6.0)	
Other	168 (6.5)	136 (6.5)	32 (6.7)	
White	1665 (64.6)	1365 (65.2)	300 (62.4)	
NDI quartile				0.04
1st	646 (25.1)	544 (26.0)	102 (21.2)	
2nd	635 (24.7)	515 (24.6)	120 (25.0)	
3rd	647 (25.1)	530 (25.3)	117 (24.3)	
4th	648 (25.2)	506 (24.2)	142 (29.5)	
Needs interpreter [yes]	68 (2.6)	57 (2.7)	11 (2.3)	0.59
Clinical variables				
BMI				0.33
<18.5	62 (2.4)	47 (2.2)	15 (3.1)	
18.5 to <25	874 (33.9)	706 (33.7)	168 (34.9)	
25 to <30	924 (35.9)	746 (35.6)	178 (37.0)	
≥30	716 (27.8)	596 (28.5)	120 (25.0)	
CCI				0.63
0	409 (15.9)	331 (15.8)	78 (16.2)	
1–2	1327 (51.5)	1086 (51.8)	241 (50.1)	
3–4	571 (22.2)	455 (21.7)	116 (24.1)	
5+	269 (10.4)	223 (10.6)	46 (9.6)	
Smoking history				0.001
Yes	1997 (77.5)	1592 (76.0)	405 (84.2)	
No	577 (22.5)	503 (24.0)	76 (15.8)	
Cancer variables				
Histology				< 0.001
Adenocarcinoma	1873 (72.7)	1582 (75.5)	291 (60.5)	
Squamous cell carcinoma	451 (17.5)	316 (15.1)	135 (28.1)	
Other	252 (9.8)	197 (9.4)	55 (11.4)	

Data are expressed as *n* (%) or median [IQR]

*IQR* interquartile range, *NDI* neighborhood deprivation index, *BMI* body mass index, *CCI* Charlson comorbidity index

Most patients did not undergo preoperative invasive nodal staging (81.3%). Of the 481 patients who underwent invasive nodal staging, 32.0% (*n* = 154) underwent EBUS and 68.0% (*n* = 327) underwent mediastinoscopy. Furthermore, 59.3% (*n* = 285) of patients underwent invasive nodal staging in advance of their surgery, while 40.7% (*n* = 196) underwent invasive nodal staging on the same day. Patients who underwent preoperative invasive nodal staging were more commonly male, Black, and from more deprived neighborhoods. They were more likely to have a smoking history and squamous cell carcinoma. Clinical N1 (10.6% vs. 2.6%) and IB+ (54.1% vs. 25.7%) tumors were more common in patients who had undergone invasive nodal staging than those who had not (Table 2). Similarly, these patients also had higher pathologic N and overall TNM stages.

**Table 2:** Staging covariates by receipt of preoperative invasive nodal staging, all patients

	All [*N* = 2576]	No preoperative nodal staging [*n* = 2095]	Preoperative nodal staging [*n = *481]	*p*-Value
Clinical stage				
Nodal stage				< 0.001
N0	2449 (95.1)	2025 (96.7)	424 (88.2)	
N1	106 (4.1)	55 (2.6)	51 (10.6)	
Nx	21 (0.8)	15 (0.7)	6 (1.3)	
Overall cTNM stage				< 0.001
IA	1758 (68.3)	1543 (73.7)	215 (44.7)	
IB	499 (19.4)	363 (17.3)	136 (28.3)	
IIA	188 (7.3)	112 (5.4)	76 (15.8)	
IIB	110 (4.3)	62 (3.0)	48 (10.0)	
Undecided	21 (0.8)	15 (0.7)	6 (1.3)	
Surgical variables				
Surgery type				< 0.001
Wedge	330 (12.8)	299 (14.3)	31 (6.4)	
Segmentectomy	59 (2.3)	55 (2.6)	4 (0.8)	
Lobectomy	2144 (83.2)	1726 (82.4)	418 (86.9)	
Bilobectomy	14 (0.5)	7 (0.3)	7 (1.5)	
Pneumonectomy	29 (1.1)	8 (0.4)	21 (4.4)	
Surgical lymph node sampling quality				
≥1 surgical nodes sampled	2508 (97.4)	2028 (96.8)	480 (99.8)	< 0.001
No. of surgical nodes sampled	6 [3-11]	6 [3–10]	9 [4–14]	0.001
Surgical samples meeting 3N2 1N1	1128 (43.8)	857 (40.9)	271 (56.3)	< 0.001
Pathologic stage				
Nodal stage				< 0.001
N0	2279 (88.5)	1884 (89.9)	395 (82.1)	
N1	214 (8.3)	136 (6.5)	78 (16.2)	
N2	9 (0.3)	4 (0.2)	5 (1.0)	
Nx	74 (2.9)	71 (3.4)	3 (0.6)	
Overall pTNM stage				< 0.001
IA	1311 (50.9)	1162 (55.5)	149 (31.0)	
IB	665 (25.8)	529 (25.3)	136 (28.3)	
IIA	277 (10.8)	178 (8.5)	99 (20.6)	
IIB	236 (9.2)	147 (7.0)	89 (18.5)	
IIIA	9 (0.4)	4 (0.2)	5 (1.0)	
IIIB	0 (0.0)	0 (0.0)	0 (0.0)	
IV	4 (0.2)	4 (0.2)	0 (0.0)	
Undecided	74 (2.9)	71 (3.4)	3 (0.6)	

Data are expressed as *n* (%) or median [IQR]

*IQR* interquartile range

Patients with invasive nodal staging were more likely to have had more extensive surgery, at least one lymph node sampled surgically, a higher median number of nodes sampled (9 [IQR 4–14] vs. 6 [IQR 3–10]), and a higher proportion of surgical samples meeting CoC criteria (3N2 1N1: 56.3% vs. 40.9%). Nodal counts were lower in patients who underwent in-advance invasive nodal staging versus same day; lymph nodes obtained during the same anesthetic procedure were included in the surgical pathology report. Only 47.4% (*n* = 135) of patients who underwent invasive nodal staging in advance of surgery had nodal sampling that met CoC criteria, compared with 69.4% (*n* = 136) of same-day patients. Similar trends were noted for median surgical lymph node counts (in advance: 8 [IQR: 3-12] vs. same day: 10.5 [IQR 6–16]).

Among all patients, nodal (N) status was more likely to change postoperatively in patients with invasive nodal staging than in those without invasive staging (upstaged: 10.4% vs. 5.3%; downstaged: 4.0% vs. 1.3%; *p* < 0.001) (Table 3). These clinical to pathologic stage change trends were consistent even when only considering patients whose surgical specimens met CoC criteria (upstaged: 10.0% vs. 6.9%; downstaged: 4.1% vs. 1.5%; *p* = 0.02)). More detailed nodal stage change data are available in electronic supplementary material (ESM) Table 1. For TNM stage, patients with invasive nodal staging were also more likely to be upstaged (30.6% vs. 23.6%) and downstaged (10.4% vs. 4.3%) [*p* < 0.001].

**Table 3:** Clinical to pathologic stage change, all patients

	All patients				Surgical specimens meeting 3N2 1N1			
	All	No preoperative nodal staging	Preoperative nodal staging	*p*-value	All	No preoperative nodal staging	Preoperative nodal staging	*p*-value
All patients	*N* = 2576	*n* = 2095	*n* = 481		*N* = 1128	*n* = 857	*n* = 271	
Nodal stage change				< 0.001				0.02
Upstaged	160 (6.2)	110 (5.3)	50 (10.4)		86 (7.6)	59 (6.9)	27 (10.0)	
Downstaged	46 (1.8)	27 (1.3)	19 (4.0)		24 (2.1)	13 (1.5)	11 (4.1)	
No change	2276 (88.4)	1873 (89.4)	403 (83.8)		1003 (88.9)	772 (90.1)	231 (85.2)	
Undecided	94 (3.7)	85 (4.1)	9 (1.9)		15 (1.3)	13 (1.5)	2 (0.7)	
TNM stage change				< 0.001				0.02
Upstaged	642 (24.9)	495 (23.6)	147 (30.6)		288 (25.5)	221 (25.8)	67 (24.7)	
Downstaged	140 (5.4)	90 (4.3)	50 (10.4)		70 (6.2)	43 (5.0)	27 (10.0)	
No change	1700 (66.0)	1425 (68.0)	275 (57.2)		755 (66.9)	580 (67.7)	175 (64.6)	
Undecided	94 (3.7)	85 (4.1)	9 (1.9)		15 (1.3)	13 (1.5)	2 (0.7)	
*All nodal upstaged patients*	*N* = 160	*n* = 110	*n* = 50		*N* = 86	*n* = 59	*n* = 27	
Node change relevance				0.14				1.0
<N2	151 (94.4)	106 (96.4)	45 (90.0)		80 (93.0)	55 (93.2)	25 (92.6)	
N2	9 (5.6)	4 (3.6)	5 (10.0)		6 (7.0)	4 (6.8)	2 (7.4)	
All TNM upstaged patients	*N* = 642	*n* = 495	*n* = 147		*N* = 288	*n* = 221	*n* = 67	
TNM stage change relevance				0.18				1.0
<IIIA	629 (98.0)	487 (98.4)	142 (96.6)		279 (97.6)	214 (96.8)	65 (97.0)	
IIIA+	13 (2.0)	8 (1.6)	5 (3.4)		9 (3.1)	7 (3.2)	2 (3.0)	

Data are expressed as *n* (%)

Of the 160 patients who were node-upstaged, only 9 (5.6%) were ‘substantially upstaged’ to N2. Similarly, of the 642 patients who were TNM-upstaged, only 13 (2.0%) were upstaged to IIIA or greater. For the 8 patients without invasive nodal staging who were upstaged to IIIA+, 4 were upstaged to IIIA because of their nodal status (cN0 > pN2) and the remaining 4 were upstaged to IIIB+ because of tumor size. There was no difference in significant N or TNM upstaging based on receipt of preoperative invasive nodal staging (*p* = 0.14 and *p* = 0.18, respectively). When considering these outcomes in the overall study population, they were rare (N2: 0.3%; IIIA+: 0.5%).

Over two-thirds of clinical IB+ patients did not undergo preoperative invasive nodal staging (66.8%). Patients who underwent preoperative invasive nodal staging were more likely to be from more deprived neighborhoods and have squamous cell histology, clinical N1 disease, and clinical TNM stage II disease (ESM Table 2). They were also more likely to have undergone more extensive resections and have more lymph nodes in their surgical pathology reports. There was no difference in nodal or TNM upstaging or downstaging for clinical IB+ patients based on receipt or non-receipt of invasive nodal staging (Table 4). There was still no difference in nodal or TNM upstaging when only considering patients whose nodal specimens met CoC criteria. Among all upstaged clinical IB+ patients, 5 were upstaged to N2 and 7 were upstaged to IIIA+. There was no difference in ‘substantial upstaging’ (N or TNM) based on receipt of invasive nodal staging, and this was consistent for patients whose surgical nodal samples met the CoC criteria.

**Table 4:** Clinical to pathologic stage change, clinical 1B+ patients

	All patients				Surgical specimens meeting 3N2 1N1			
	All	No preoperative nodal staging	Preoperative nodal staging	*p*-Value	All	No preoperative nodal staging	Preoperative nodal staging	*p*-Value
All clinical IB+	*N* = 781	*n* = 522	*n* = 259		*N* = 429	*n* = 267	*n* = 162	
Nodal stage change				0.43				0.69
Upstaged	59 (7.6)	38 (7.3)	21 (8.1)		31 (7.2)	20 (7.5)	11 (6.8)	
Downstaged	46 (5.9)	27 (5.2)	19 (7.3)		24 (5.6)	13 (4.9)	11 (6.8)	
No change	676 (86.6)	457 (87.6)	219 (84.6)		374 (87.2)	234 (87.6)	140 (86.4)	
TNM stage change				0.65				0.88
Upstaged	159 (20.4)	104 (19.9)	55 (21.2)		74 (17.3)	48 (18.0)	26 (16.1)	
Downstaged	140 (17.9)	90 (17.2)	50 (19.3)		70 (16.3)	43 (16.1)	27 (16.7)	
No change	482 (61.7)	328 (62.8)	154 (59.5)		285 (66.4)	176 (65.9)	109 (67.3)	
All nodal upstaged clinical 1B+	*N* = 59	*n* = 38	*n* = 21		*N* = 31	*n* = 20	*n* = 11	
Node change relevance				0.05				0.28
<N2	54 (91.5)	37 (97.4)	17 (81.0)		28 (90.3)	19 (95.0)	9 (81.8)	
N2	5 (8.5)	1 (2.6)	4 (19.1)		3 (9.7)	1 ( 5.0)	2 (18.2)	
All upstaged clinical IB+	*N* = 159	*n* = 104	*n* = 55		*N* = 74	*n* = 48	*n* = 26	
TNM stage change relevance				0.24				0.61
<IIIA	152 (95.6)	101 (97.1)	51 (92.7)		70 (94.6)	46 (95.8)	24 (92.3)	
IIIA+	7 (4.4)	3 (2.9)	4 (7.3)		4 (5.4)	2 (4.2)	2 (7.7)	

Data are expressed as *n* (%)

## Discussion

As imaging sensitivity improves and NSCLC is detected at earlier stages, it is not clear that all early-stage NSCLC surgical candidates require invasive nodal staging as part of their preoperative staging work-up. Accurate pathologic staging ultimately leads to improved survival, presumably through more stage-appropriate treatment,^19^ but there may be some early-stage patients in whom high-quality intraoperative nodal staging is sufficient. Even among clinical 1B+ patients (in whom invasive nodal staging is recommended) with surgical nodal specimens meeting 3N2 1N1, there was no association with nodal stage change based on receipt of invasive nodal staging.

Our institution generally only performs invasive nodal staging for suspected early-stage NSCLC if there is a clear nodal radiographic indication (node > 1.0 cm short axis, SUV > 3.0) or a large tumor (> 4.0 cm, irrespective of location), and significant clinical judgment is allowed. Only 0.3% of the 2576 patients were upstaged to N2 and only 0.5% were upstaged to IIIA+. Among the IIIA+ patients, the majority were upstaged to pathologic stage IIIA for whom appropriate treatment sequencing is debated. Importantly, there was no difference in ‘substantial upstaging’ based on receipt of invasive nodal staging. Our results are not prescriptive; they do not clearly define who should receive invasive nodal staging. However, the low rate of overall pathologic upstaging to N2 or IIIA+ highlights the potential adequacy of the existing criteria at our institution for early-stage lung cancer patients.

Current invasive nodal staging recommendations may be overly broad because of advancements in imaging quality, resulting in a low pretest probability of mediastinal disease in many early-stage NSCLC patients. Imaging technology, from the acquisition to reconstruction of images, has steadily improved.^20^ Integrated PET/CT was only introduced into clinical practice in 1998.^21^ Recent oncologic studies indicate improved sensitivity and specificity for lymph node metastasis with newer PET/CT technology.^22^ Increased imaging accuracy and staging precision have also driven renewed interest in sublobar resection for select early-stage NSCLC.^23^ In a meta-analysis of NSCLC patients diagnosed between 1980 and 2011, PET sensitivity for mediastinal nodal disease in all clinical stages was 80%.^8^ In 2012, the negative predictive value of PET/CT for mediastinal disease in imaging-based clinical stage I (cT1-2N0) disease was reportedly 93%;^6^ however, more recently, PET/CT was demonstrated to have a 97.4% sensitivity and 97.8% negative predictive value for mediastinal disease.^24^ As imaging sensitivity improves, the pretest probability of unidentified mediastinal nodal disease shrinks. In addition to a decreased pretest probability of mediastinal disease with higher-quality imaging, invasive nodal staging sensitivity is also lower in earlier-stage disease. EBUS sensitivity for N2 disease in imaging-based N0–N1 disease is only 38–53%,^12–14^ while mediastinoscopy sensitivity for N2 disease is 47% in suspected N0 disease^8^ and 73% for suspected N1 disease.^15^ Additionally, EBUS and mediastinoscopy sensitivity depend heavily on the operator and the location of suspected nodal involvement; almost half of false negatives are due to inaccessible location.^8^ Preoperative invasive nodal staging recommendations may consider the pretest probability of mediastinal nodal involvement and the test sensitivity, especially based on tumor location, more carefully.

Our study found a low rate of unexpected N2 disease (0.3%) on surgical pathology compared with prior studies. Unexpected N2 disease in imaging-designated clinical stage I (N0) has been reported at approximately 5–15%,^8,25–29^ but data are predominantly from the early 2000s, shortly after the introduction of PET/CT. Low pathologic N2 rates could hypothetically result from inadequate N2 surgical sampling. However, 97.4% of our patients had at least one lymph node sampled with a median of six nodes (IQR 3–11) and 43.8% of patients had surgical nodal samples meeting current CoC criteria between 2009 and 2019. This rate of sampling is superior to current nodal sampling practices at a comparable time period, with US studies indicating that a minority of resections meet CoC criteria (36.8%,^19^ <25.9%^30^). Among the 1128 patients in our study with adequate surgical nodal samples by CoC guidelines, only 6 (0.5%) were upstaged to N2. Even when only considering patients with adequate surgical lymph node harvests by expert committee guidelines, unexpected N2 rates in this study are still low, highlighting the adequacy of our institutional staging guidelines and suggesting enhanced imaging-based nodal staging accuracy.

Our ability to draw further conclusions about invasive nodal staging criteria is limited by the study’s inclusion and exclusion criteria; this study does not include patients of all stages who underwent invasive nodal staging. Specifically, our study population does not include patients who underwent invasive nodal staging (generally, imaging-based, node-positive disease and/or tumors >4 cm) and were appropriately staged as clinical IIIA+. However, we further analyzed a subset of these clinically stage IIIA+ patients and nearly all were already clinical stage III+ based on imaging alone, given imaging sensitivity. Based on institutional recommendations, tumors >4 cm (T2) or N1 on imaging are particularly liable to be redefined as clinical IIIA+ on the basis of invasive nodal staging; N2 tumors on imaging are already IIIA+. Conversely, patients who had node-positive disease, particularly N2 disease, on imaging but whose clinical stage was revised to <IIIA after invasive nodal staging were included. In addition, we were not able to thoroughly evaluate the clinician decisions behind deciding on whether or not to perform preoperative invasive nodal staging. Finally, our sample inherently excludes patients whose surgery was aborted after intraoperative discovery of extensive, unexpected mediastinal disease, since these patients did not undergo surgery with curative intent; however, this is a rare outcome because resection is generally completed at our institution in the setting of limited mediastinal disease. Based on the aforementioned limitations, we cannot make prescriptive recommendations about who should receive invasive nodal staging, but we can conclude that our current narrower institutional nodal staging recommendations did not result in high rates of unexpected N2 disease. Furthermore, although patients who do not receive invasive nodal staging could be at a higher risk of unexpected N2, we did not find that lack of invasive nodal staging was associated with higher rates of unexpected N2.

Our results are relatively generalizable to early-stage NSCLC patients in the US. Our study population had a similar proportion of stage II disease and a lower proportion of squamous cell histology compared with patients in the US with NSCLC. SEER registry (stage II: 22.2% of early-stage NSCLC; squamous cell histology: 29.5%).^31^ While squamous cell cancers tend to be more centrally located, adenocarcinomas are more likely to have unexpected N2.^9^ Our study lacks important information on imaging characteristics, such as ground-glass or solid, and tumor location, particularly centrality, which is a common issue in similar studies.^3^

However, our study has key attributes that are unique for a multicenter study. In particular, our study benefits from institution-wide staging recommendations that are harmonized across all 21 hospitals. In our study, EBUS availability and quality is more representative of the community experience, although limited EBUS availability has also been noted to delay care in the academic setting.^32^ Centralization and regionalization of many facets of thoracic oncology care in our integrated healthcare system affords us greater insight into the clinical gestalt driving invasive nodal staging decisions. Anecdotally, centrality and slightly increased size (>3 cm) are common reasons that clinicians do not adhere to guidelines.

Guidelines for NSCLC management are rapidly evolving. Accurate preoperative mediastinal staging has been essential because tumor resectability and treatment sequencing diverge at this point; treatment algorithms have historically recommended upfront surgical resection in fit candidates until N2 involvement;^5^ However, the 5-year survival of upfront resected unexpected N2 has been found to be similar to resected clinical N1 disease treated with adjuvant therapy.^9,12^ N2 disease is more heterogenous than previously thought,^4^ and there is increasing evidence that select N2 disease benefits from resection.^33,34^ Recent immunotherapy trial results with neoadjuvant chemoimmunotherapy in clinical IB–IIIA also challenge whether mediastinal nodal involvement is the fulcrum for upfront resection.^35,36^ With the success of adjuvant immunotherapy and the absence of head-to-head trials, it is unclear if the treatment sequencing (neoadjuvant vs. adjuvant) is a defining component of these regimens’ favorable outcomes.^10,37^ In the background, imaging availability and quality have steadily improved.^38^ To avoid subjecting large proportions of early-stage NSCLC patients to potential delays in care and invasive procedures with limited benefit, further study is needed to redefine the exact imaging-based tumor features that necessitate invasive nodal staging. Risk prediction models that incorporate imaging findings with a minimum mediastinal metastasis pretest probability can help.^39^

## Conclusion

Although 81.3% of early-stage NSCLC patients did not undergo preoperative invasive nodal sampling and 97.4% had nodes sampled at resection, < 1% were upstaged to N2 after surgery. There was no association between receipt of preoperative invasive nodal staging and N2 upstaging. Receipt of preoperative invasive nodal staging for selected early-stage NSCLC patients in our integrated health system did not result in substantial N2 nodal upstaging. We believe these results from our resected early-stage NSCLC cohort can add to future prospective studies and assist guideline committee recommendations to determine an even more precise group of patients where preoperative invasive nodal staging may be recommended.

## Supplementary Information

Below is the link to the electronic supplementary material.

Supplementary file1 (XLSX 14 KB)
